# Evidence for Horizontal Transmission and Recirculation of Shiga Toxin-Producing *Escherichia coli* in the Beef Production Chain in South Africa Using Whole Genome Sequencing

**DOI:** 10.3390/pathogens13090732

**Published:** 2024-08-29

**Authors:** Libby Obumneke Onyeka, Abiodun A. Adesiyun, Arshad Ismail, Mushal Allam, Karen H. Keddy, Peter N. Thompson

**Affiliations:** 1 Department of Production Animal Studies, Faculty of Veterinary Science, University of Pretoria, Onderstepoort 0110, South Africa; aadesiyun@gmail.com; 2 Department of Veterinary Public Health and Preventive Medicine, College of Veterinary Medicine, Michael Okpara University of Agriculture, Umudike 440101, Abia State, Nigeria; 3 Department of Basic Veterinary Sciences, School of Veterinary Medicine, Faculty of Medical Sciences, University of the West Indies, St. Augustine 999183, Trinidad and Tobago; 4 Sequencing Core Facility, National Institute for Communicable Diseases a Division of the National Health Laboratory Service, Johannesburg 2192, South Africa; arshadi@nicd.ac.za (A.I.); mushalallam@gmail.com (M.A.); 5 Department of Biochemistry and Microbiology, Faculty of Science, Engineering and Agriculture, University of Venda, Thohoyandou 0950, South Africa; 6 Institute for Water and Wastewater Technology, Durban University of Technology, Durban 4000, South Africa; 7 Department of Genetics and Genomics, College of Medicine and Health Sciences, United Arab Emirates University, Al Ain 15551, United Arab Emirates; 8 Department of Veterinary Tropical Diseases, Faculty of Veterinary Science, University of Pretoria, Onderstepoort 0110, South Africa; karen.keddy@up.ac.za

**Keywords:** horizontal transmission, multilocus sequence typing, serogenotype, antimicrobial resistance genes, South Africa

## Abstract

We used whole genome sequencing (WGS) as an epidemiologic surveillance tool to elucidate the transmission dynamics of Shiga toxin-producing *Escherichia coli* (STEC) strains along the beef production chain in South Africa. Isolates were obtained from a cattle farm, abattoirs and retail outlets. Isolates were analysed using WGS on a MiSeq platform (Illumina, San Diego, CA, USA) and phylogenetic analysis was carried out. Of the 85 isolates, 39% (33) carried the *stx* gene and 61% (52) had lost the *stx* gene. The prevalence of *stx* subtypes was as follows; *stx*_1*a*_ 55% (18/33), *stx*_1*b*_ 52% (17/33), *stx*_2*a*_ 55% (18/33), *stx*_2*b*_ 27% (9/33), *stx*_2*dB*_ 30% (10/33) and *stx*_2*d*1*A*_ 15% (5/33). Thirty-five different serogenotypes were detected, of which 65% (56) were flagellar H-antigens and 34% (29) were both O-antigens and flagellar H-antigens. We identified 50 different sequence types (STs), and only nine of the isolates were assigned to three different clonal complexes. Core genome phylogenetic analysis revealed genetic relatedness, and isolates clustered mainly according to their STs and serogenotypes regardless of *stx* subtypes. This study provides evidence of horizontal transmission and recirculation of STEC strains in Gauteng province and demonstrates that every stage of the beef production chain plays a significant role in STEC entry into the food chain.

## 1. Introduction

Shiga toxin-producing *Escherichia coli* (STEC) is a food- and water-borne pathogen reported in numerous outbreaks worldwide [1]. STEC causes a broad spectrum of disease ranging from mild to severe bloody diarrhoea (haemorrhagic colitis; HC), and in some cases (5–10%) it can progress to haemolytic uraemic syndrome (HUS) [1,2]. The ability of STEC to cause human disease is influenced primarily by the production of Shiga-like toxins (*stx*), which are encoded by *stx* genes carried on bacteriophages [2,3]. The *stx* genes are classified into two major types, *stx*_1_ and *stx*_2_ [3]. Scheutz et al. [3] proposed a subtype classification of the two major *stx* variants: *stx*_1_ which consists of *stx*_1*a*_, *stx*_1*b*_, *stx*_1*c*_ and *stx*_1*d*_ and *stx*_2_ consisting of seven distinct variants, namely *stx*_2*a*_, *stx*_2*b*_, *stx*_2*c*_, *stx*_2*d*_, *stx*_2*e*_, *stx*_2*f*_ and *stx*_2*g*_. Reports have shown that *stx*_2*a*_, *stx*_2*c*_ and *stx*_2*d*_ subtypes are associated with the development of HC and HUS [3]. A group of virulence factors encoded by a chromosomal region, described as the 35-kb locus of enterocyte effacement (LEE) pathogenicity island (PAI), confer the attaching and effacing (A/E) phenotype for STEC [4]. The STEC LEE comprises genes encoding intimin, an adhesion factor (*eaeA*), the translocated receptor of intimin (*tir*), the secreted proteins *EspA*, *EspB* and *EspD* and the type III secretion pathway [5]. Other potential and putative virulence factors of pathogenic *E. coli*, including a wide array of adhesins, toxins, siderophores and secretion systems, empower the organism to colonize the intestinal epithelium, evade or manipulate host defence mechanisms, provoke harmful inflammatory reactions within the host and inflict direct harm upon host cells and tissues [6].

Over 100 O-serotypes of the more than 470 known STEC serotypes have been associated with human disease [6]. STEC O157:H7 has gained notoriety in major foodborne outbreaks and sporadic cases worldwide including in the USA, Canada, Japan and the United Kingdom [7]. However, several non-O157 STEC strains have been frequently linked with HC and HUS, predominantly strains of several serogroups—O26, O45, O103, O111, O121 and O145, termed the “big six” [1]. Non-O157 STEC strains are reported to cause more infections than do O157:H7 strains in Europe [7], including the 2011 Germany and France outbreak of O104:H4 enteroaggregative STEC [8].

The hind gut of cattle is recognised as the main asymptomatic reservoir and has the capacity to shed STEC transiently [9]. The pathogen’s excretion rates and concentration in faeces contribute considerably to its epidemiology and transmission within herds and in humans [10]. If the pathogen load in cattle entering the abattoir is high, then the likelihood of carcass contamination in the beef production chain is increased [11]. As such, the cattle farm plays a vital role in the beef chain. Additionally, STEC strains have been associated with human disease through the consumption of undercooked beef or beef-based products [12,13], which have been contaminated by cattle faeces during slaughter or processing as a result of cross-contamination, mainly from the hide and occasionally from gut contents [14,15]. In addition to cattle farms and abattoirs, meat retail outlets play an important role in the transmission of STEC-contaminated raw beef and ready-to-eat (RTE) beef products [16]. Contamination could arise at several stages, such as during meat cutting and further processing, such as with mincemeat or sausages made from mincemeat. A few colonized livestock or contaminated carcasses could contaminate a large quantity of ground beef [17]. Consequently, the presence of STEC throughout the beef production chain is a potential public health hazard.

In South Africa, although a few studies have reported the prevalence and virulence profiles of bovine STEC isolates [18,19], little has been done using whole genome sequencing (WGS) as a subtyping method for bovine isolates recovered along the beef chain. This study aimed to apply WGS as a molecular subtyping method to assess the virulence potential, phylogenetic relationships and diversity of STEC isolates recovered along the beef chain in Gauteng, the most densely populated province of South Africa.

## 2. Materials and Methods

### 2.1. Sources of Isolates

The STEC isolates in this study were recovered from three sources as previously described in Onyeka et al. [20,21,22]. 

#### 2.1.1. Cattle Feedlots

Isolates (*n* = 30) were recovered from a longitudinal study conducted between September 2016 and February 2017, which determined the presence of shedders and super-shedders in a feedlot cattle operation in northern Gauteng, South Africa. Faecal samples were collected by rectal grab from randomly selected individual animals [23].

#### 2.1.2. Abattoir and Retail Outlets

During November 2015 to November 2016, a random cross-sectional survey investigated STEC prevalence and molecular characteristics on beef carcasses and in beef products in Gauteng. For the abattoir study, 12 abattoirs located in Gauteng North (5), Gauteng East (4) and Gauteng West (3) were selected for the survey. Individual animals and carcasses were tagged and tracked in simple or continuous slaughter lines, and samples were obtained at different point locations in processing plants. From the abattoir study, 28 isolates were recovered. In addition, 7 isolates were recovered from tagged cattle followed from the farm to the abattoir in February for slaughter. The samples used in this study comprised carcass swabs in swab rinse kit solution (SRK), swabs from perineum hide swabs, swabs from walls and floor, faeces, rinsates and abattoir effluents [24].

For the retail study, 31 retail outlets (large chain supermarkets and butcheries) located across northern Gauteng were sampled by purchasing five different raw beef and ready-to-eat beef products. The samples comprised raw beef including brisket, mincemeat and boerewors and beef-based RTE products including biltong and cold meat and were sampled from the retail outlets during four seasons: summer, autumn, winter and spring. A total of 21 isolates were recovered from the retail outlets [20].

#### 2.1.3. Isolation of STEC Strains

Only enrichment broth cultures that were PCR-positive for *stx*_1_, *stx*_2_ or both were considered positive for STEC and were cultured to isolate STEC strains. To isolate O157 STEC the procedure consisted of immunomagnetic separation (IMS) assays using Dynabeads^®^ anti-*E. coli* O157 (Thermo Fisher Scientific, Waltham, USA), as recommended by the manufacturer. The immune-concentrated bacterial suspensions were then inoculated onto sorbitol with MacConkey agar (SMAC) supplemented with potassium tellurite 2.5 mg/L and cefixime 0.05 mg/L (Himedia Laboratories Pvt., Thane, India). Likewise, 10 μL of enriched broth sample was streaked on a chromogenic agar, CHROMagar O157 (CHROMagar Microbiology, Paris, France) supplemented with potassium tellurite 2.5 mg/L and cefixime 0.05 mg/L (Himedia Laboratories Pvt., Thane, India). Subsequently, the plates were incubated for 24–30 h at 37 °C, and up to seven suspect colonies with different phenotypes were picked from each plate and tested by latex agglutination (Welcolex^®^ *E. coli* O157 Rapid latex agglutination test, Remel, Leicestershire, UK). Enriched control strain—*E. coli* ATCC 43888 (O157:H7)—was also inoculated for phenotypic control and assessment.

To isolate non-O157 STEC, 10 μL of enriched broth sample was streaked on MacConkey agar containing crystal violet and salt and onto CHROMagar STEC (CHROMagar Microbiology, Paris, France). The plates were incubated for 24–30 h at 37 °C, and representative suspect colonies were subcultured on nutrient agar plates for further biochemical testing. For further biochemical confirmation, isolates were randomly selected and confirmed as *E. coli* using the bioMérieux Vitek 2 Compact system (BioMérieux, Marcy l’Étoile, France).

#### 2.1.4. Multiplex PCR to Identify Virulence

A DNA template of STEC isolates was prepared using the QIAamp DNA Stool Mini Kit (Qiagen, Hilden, Germany), following the manufacturer’s instructions. The DNA templates were investigated for the presence of *stx*_1_, *stx*_2_, *eaeA* and *hlyA* genes using mPCR as described by Paton and Paton [25] and Lindsey et al. [26], with slight modifications. All the isolates positive for the presence of toxin genes were stored at −20 °C until subjected to analysis.

#### 2.1.5. Validation of mPCR

The assay conditions were optimized using molecular control strains obtained from the National Institute for Communicable Diseases—Centre for Enteric Diseases, South Africa (2014-2015 VTEC EQA—*E. coli* RR18-3022 O157, *eaeA*, *stx*_1*a*_, *stx*_2*a*_) and the enrichment control strain *E. coli* ATCC 43888 (O157:H7) *stx*_1_. The mPCR was validated by Sanger sequencing of PCR products.

### 2.2. Whole Genome Sequencing and Analysis

The Nextera XT DNA library prep kit was used to prepare paired-end libraries for 85 genomic DNA isolates, followed by 2 × 300-bp sequencing on a MiSeq platform (Illumina, Inc., San Diego, CA, USA) aiming at a coverage of at least 100-fold. The resultant paired-end reads were checked for quality control (QC) of average Q-score > 20 and trimmed using FASTP version 0.19.5 [27]. The sequence reads were de novo assembled using SKESA version 2.4.0 [28]. Gene annotation of all contiguous sequences (contigs) was carried out using Prokka [29]. Multilocus sequence typing (MLST) using the seven conserved housekeeping genes of *E. coli* scheme 1 was determined using Seemann T, mlst Github (https://github.com/tseemann/mlst, accessed on 20 March 2023). ABRicate [30] and subsequently ECtyper [31], was used for in silico serotyping of *E. coli*. Comprehensive antibiotic resistance database (CARD) was used for antimicrobial resistance genes [32], and *E. coli* virulence factors were determined using known virulence factors obtained from the virulence factor database (VFDB) [33].

### 2.3. Phylogenetic Analysis of STEC Isolates

A rapid, large-scale prokaryote pan-genome analysis pipeline (Roary) was used to determine genetic relationships among the STEC genomes [34], and randomized accelerated maximum-likelihood (RAxML) analysis [35] was used to reconstruct a maximum-likelihood phylogenetic tree based on core genome single nucleotide polymorphisms (SNPs). To extract predicted coding regions from Prokka-annotated assemblies and convert them to protein sequences, the core genome alignment module in Roary was employed [32]. BlastP (https://blast.ncbi.nlm.nih.gov, accessed on 15 March 2023) was used to compare all protein sequences with one another. Proteins that had alignment similarity of ≥70% and were present in at least 90% of the isolates were defined as the core genome. RAxML [17] was used to create a bootstrapped maximum-likelihood phylogenetic tree from the resulting core genome alignment and visualized and annotated in FigTree v1.4.3 (http://tree.bio.ed.ac.uk/software/figtree/, accessed on 30 March 2023). See Supplementary Table S1 for information on the 85 isolates used in this study, NCBI accession numbers and corresponding URLs.

## 3. Results

### 3.1. Isolates

Of the 85 PCR-confirmed STEC isolates submitted for WGS, only 39% (33) harboured the *stx* gene, and 61% (52) had lost the *stx* gene. Of the 52, 85% (44) harboured the *eae* gene, and 15% (8) lacked the *eae* gene but harboured other adherence genes (Supplementary Table S2). The prevalence of *stx* subtypes was as follows: *stx*_1*a*_ 55% (18/33), *stx*_1*b*_ 52% (17/33), *stx*_2*a*_ 55% (18/33), *stx*_2*b*_ 27% (9/33), *stx*_2*dB*_ 30% (10/33) and *stx*_2*d*1*A*_ 15% (5/33) (Table 1). Thirty-five different serogenotypes, with two novel serogenotypes, were found among the 85 isolates, of which 65% (56/85) were flagellar H-antigens with O-antigen untypeable, and 34% (29/85) were both O-antigens and flagellar H-antigens including O8:H19. The *stx*_2*d*_ toxin defined by Scheutz et al. [3], namely *stx*_2*d*1*A*_, was found in five isolates, whereas *stx*_2*dB*_ was found in ten isolates. Fourteen different *stx* subtype combinations were found among the 33 isolates (Table 1). 

### 3.2. Multilocus Sequence Typing

We identified 50 different sequence types (STs), including five isolates of novel STs and three of unknown STs, among the 85 isolates. The most frequent STs were ST306 (5/85; 6%) and Novel (5/85; 6%). Only nine of the isolates were assigned to three different clonal complexes (ST that matched the central genotype at five or six loci), the remaining STs identified in this study could not be assigned to any clonal complex (Table 2).

### 3.3. Virulence Genes

**Figure 1:**
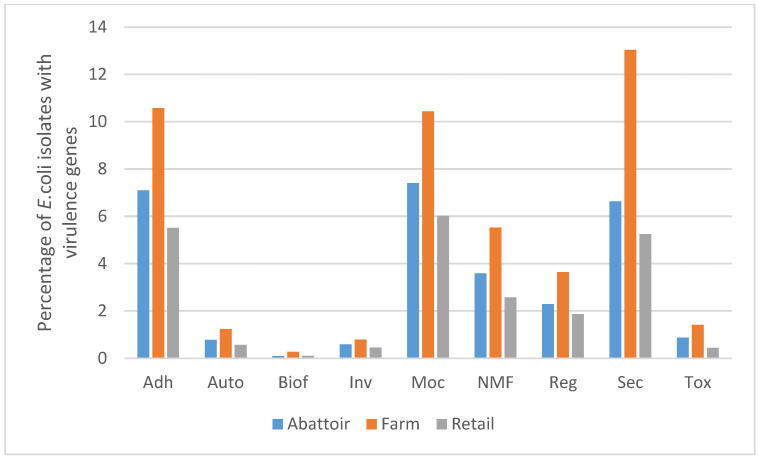
Bar-chart showing the percentage of 85 *E.coli* isolates from beef abattoirs, feedlot and retail outlets (raw beef and ready-to-eat beef products) that possessed virulence genes with their manually annotated functions in Gauteng, South Africa. Adh: adherence, Auto: autotransporter, Biof: biofilm, Inv: invasive, MoC: motility/chemotaxis, NMF: nutritional/metabolic factor, Reg: regulatory, Sec: secretory, Tox: toxin.

A total of 552 putative virulence genes were determined (Supplementary Table S2). The genes included adherence, secretory (type II/III/IV/VI secretory system/effectors) and toxin (heat-labile/stable enterotoxin, cytolethal distending toxins, colicin, exotoxin cytotoxic necrotizing factor, haemolysin and subtilase cytotoxin), among others (Figure 1). From the 85 isolates, the prevalence of the LEE encoded genes was as follows: *eae* 19% (16), *EspA* 20% (17), *EspB* 19% (16) and *EspD* 20% (17). The prevalence of plasmid-encoded virulence-associated genes was as follows: *espP* 26% (22), *katp* 11% (9), *subA* 6% (5) and *saa* 4% (3). Others prevalences included the autotransporter proteins *ehaA* 62% (53) and *ehaB* 74% (63), a heme uptake-related gene *chuA* 6% (5) and the haemolysin gene *hlyA* 31% (26). Furthermore, among the 52 *stx*-negative isolates, the virulence factors *eae*, *tir* and *chuA* were identified in the beef chain in the farm 12% (2) and retail 20% (3) isolates, each. Catalase-peroxidase (*katP*) was found in isolates from the farm 18% (3) and retail shops 20% (3) in the beef chain. Only one isolate (perineal-PdJ2-4) harboured cytolethal distending toxins (*cdtIIIA*, *cdtIIIB* and *cdtIIIC*). Additionally, we identified a selection of virulence genes associated with a high risk of diarrhoea and severe disease in humans [19], including *aatA* 6% (5/85), *cif* 7% (6/85), *escV* 11.8% (10/85), *EspA* 20% (17/85), *nleA* 9.4% (8/85), *nleB* 13% (11/85) and *tccp* 3.5% (3/85).

### 3.4. Antimicrobial Resistance Genes

We detected 66 genes of which multidrug (MDR) efflux pump genes were the most prevalent 55% (36), including the acriflavine efflux system AcrAB-TolC (*acrA*—100%; *acrB*—96.5%; *TolC*—100%) and regulators such as *cpxA* (98.8) and *gadX* (95%) (Table 3). Of notable mention is the presence of *E. coli* ampicillin class C (*AmpC*) β-lactamase genes, detected in 97.6% (83/85) of the isolates. Interestingly, we observed a low prevalence of antimicrobial resistance genes in the WOAH-OIE [23] classified list of “veterinary critically important antimicrobial agents” in cattle, which included aminoglycosides-modifying enzymes [24], nucleotidyltransferases encoded by *aadA* (5%), *aadA2* (2%), *aadA3* (4%) and *aadA4* (1%) and phosphotransferases *aph* (6)-Id (9.4%) and *aph* (3″)-Ib (9.4%) which mediate resistance against kanamycin. Others were Fosfomycin-modifying enzymes such as metalloenzyme *FosA7* (4.7%), nonfluorinated/fluorinated phenicols genes *cmIA6* (1%) and *floR* (4%) and β-lactamases *TEM-1* (4%) and *TEM-150* (14%) (Table 3 and Supplementary Table S3).

### 3.5. Phylogenetic Analysis

**Figure 2:**
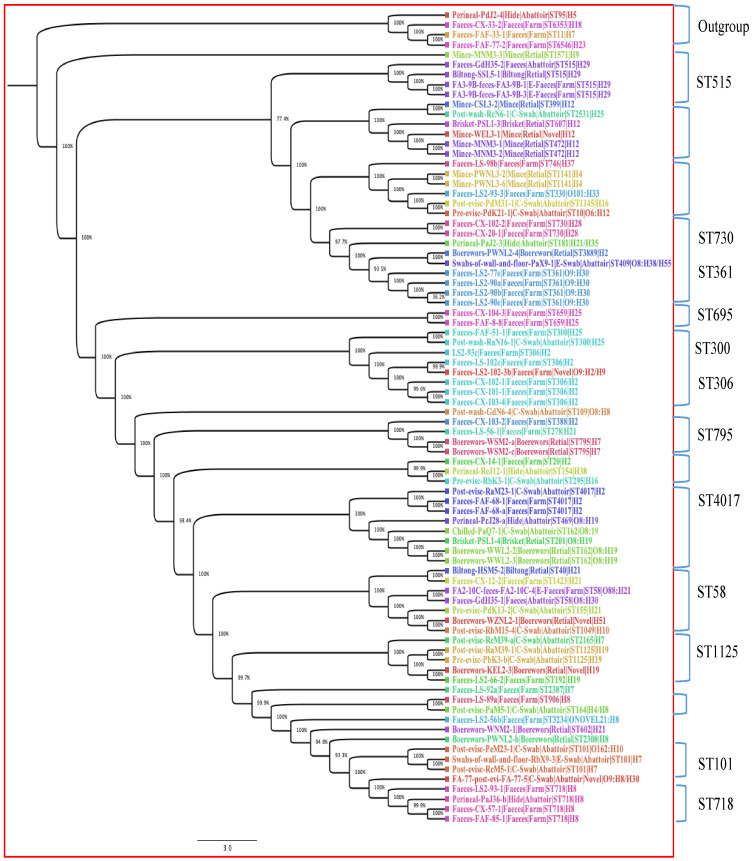
Phylogenetic tree of 82 STEC isolates based on 4760 genes (defined by core genome) from different stages in the beef production chain in Gauteng, South Africa. The node percentages are the bootstrap values from 100 replicates representing the confidence estimates of the tree topology. The scale bar indicates 10% nucleotide sequence divergence. The colours represent isolates belonging to the same sequence types.

The phylogenetic tree was built with only 82 isolates; the three isolates with unknown ST were excluded from the tree. The 82 isolates contained 4760 genes, of which 32.81% were the core genes (shared by all 82 isolates), used in constructing the tree. Core genome phylogenetic analysis revealed that isolates clustered mainly according to their STs and serogenotypes regardless of *stx* subtype. The 82 isolates were categorised into 12 clades, partly based on their STs and serogenotypes. Figure 2 shows the distribution of the 12 clades, with clades that contain similar STs being highlighted. The clade formed by ST515 belonging to serotype H29 showed a close relationship with isolates from cattle faeces (abattoir, Gauteng east), biltong (retail outlet, Gauteng north) and cattle faeces (feedlot, Gauteng north). The clade containing ST730 and ST361 showed intra-farm transmission. In addition, similar patterns of genetic relatedness were shown in isolates with ST306 (five cattle intra-farm) and ST4017 (inter-abattoir in Gauteng west and Gauteng north). However, we observed an outgroup clade including isolates from abattoir hide (ST95) and from three cattle from the farm (ST6353, ST11 and ST6546).

## 4. Discussion

In this study, we explored the potential of WGS as an epidemiologic surveillance tool to elucidate the molecular characteristics and transmission dynamics of STEC along the beef production chain (the farm-to-fork approach) in South Africa. The subtyping of *stx* genes revealed that only 39% (33) of the 85 isolates harboured the *stx* gene, and 61% (52) had lost the *stx* gene, a phenomenon termed ‘STEC lost Shiga toxin’ [36], given that our previous studies had confirmed these as STEC isolates [20,21,22]. The loss of the *stx* genes might have occurred during the initial subcultivation step or during subculturing of preserved frozen cultures to obtain genomic DNA [36,37,38]. Several studies have indicated a correlation between the loss of *stx* genes and the serotype or the specific subtype of *stx*, which are less stable in non-O157 strains [36,37,38]. Our data support these observations, since all 85 isolates were non-O157 STEC. Consequently, great caution must be exercised in the aetiological diagnosis of HC and HUS, given the possibility of a loss of *stx* genes.

Epidemiologic studies and cytotoxicity assays have revealed that the different subtypes may be associated with varying degrees of virulence or severity [1,2,39]. In this study, we detected *stx*_2*d*_ genes (*stx*_2*d*1*A*_ and *stx*_2*dB*_) and combinations of *stx*_1*a*_, *stx*_1*b*_ and *eae* (8 isolates) and *stx*_2*a*_, *stx*_2*dB*_ and *eae* (2 isolates), which have the potential to cause HC and HUS in humans [1,39,40].

In addition to the *stx* genes, we observed genes encoding 81 type III secretion systems (T3SSs). These are major virulence genes that contribute to the severity of STEC disease [32]. The presence of T3SSs in our isolates is of public health importance, as this presence in cattle populations, abattoirs and meat products in South Africa increases the risk for zoonotic, environmental and foodborne transmission of the most virulent strains [41].

From the 85 isolates, we found 35 serogenotypes of which 65% (56/85) were O-serogroup untypeable (ONT). Among the 56 flagellar antigens we identified H2, H7, H8, H12, H16, H19, H21, H25 and H28, which have been associated with pathogenic STEC [40,42]. Additionally, STEC ONT:H7 in this study harboured the highest number of virulence-associated genes linked with severe clinical symptoms (*stx*_2*dB*_, *stx*_2*a*_, *subA*, *eae*, *espP*, *hlyA*, *katP*, *tir*, *chuA* and *astA*). Other isolates which had more virulence genes included ONT:H25 (*stx*_2*dB*_, *stx*_2*a*_, *eae*, *espP*, *hlyA*, *katp*, *tir* and *astA*), ONT:H2 (*stx*_2*dB*_, *stx*_2*a*_, *espP*, *saa*, *tir* and *astA*), ONT:H8 (*stx*_2*d*1*A*_, *stx*_2*a*_, *espP*, *hlyA*, *katp* and *astA*) and O8:H19 (*stx*_2*dB*_, *stx*_2*a*_, *subA*, *espP*, *saa* and *hlyA*). Our results confirm that pathogenic *E. coli* in the beef production chain in Gauteng, South Africa comprises a genetically heterogeneous family of bacteria. Notably, O8:H19 (five isolates), ONT:H8 (six isolates) and ONT:H21(six isolates) have been linked with human disease in South Africa [41]. Furthermore, in the Netherlands and Germany, O8:H19 has been associated with HUS, while O8:H8 has been associated with mild infection [43]. STEC O8:H19 have been recovered from healthy cattle across the globe, including Europe [43], China [44] and Mexico [40].

In South Africa and other southern African countries, the importance of STEC has been highlighted by numerous clinical cases of diarrhoea in children and adults between 2006-2013 in which a diverse range of STEC serogroups (O4, O5, O8:H19, ONT:H8, ONT:H21,O21, O26, O84, O111, O113, O117 and O157) were implicated [45].

This study revealed a high prevalence of *E. coli* ampicillin class C (*AmpC*) β-lactamase genes, detected in 98% (83/85) of the isolates, clinically known to confer resistance to penicillin-like and cephalosporin-class antibiotics [46]. Our result is comparable with the findings of Iweriebor et al. [47], who reported AmpC beta-lactamases (penicillin and cephalosporin resistance) in 90% of isolates originating from two dairy cattle farms in South Africa. Additionally, multidrug efflux pumps serve as a primary defence mechanism in all bacteria, reducing the intracellular concentration of antimicrobials. A single multidrug efflux pump can expel various antibiotics, thereby contributing to bacterial pathogenicity and multidrug resistance [48]. For example, the AcrAB-TolC observed in this study is a house-keeping efflux pump which is involved in the extrusion of a wide spectrum of antibiotics including macrolides, linezolid, novobiocin, rifampin, fusidic acid, chloramphenicol, fluoroquinolone, tetracycline, nalidixic acid and β-lactam antibiotics among others [49,50]. Hence, the observed prevalence of 55% (36/66) of MDR efflux pump genes is also notable, considering that the WHO [51] has included AMR among the “top 10 threats for global health”. This trend of resistance, along with the MDR profiles, could stem from the isolate source as it originates from livestock, mainly cattle, and could be attributable to the magnitude and scale of AMR presence and persistence in the study area. South Africa and other industrializing economies such as China, Brazil, India and Russia are regarded as hotspots for antimicrobial resistance due to intensive livestock production and the concomitant increase in antibiotic use in animal husbandry [52,53].

Interestingly, the cgMLST revealed a high genomic diversity of strains, with only nine isolates grouped into three clonal complexes (Table 2). The three abattoir samples originating from three different geographic locations (Pre-evis-PdK13-2-Gauteng north, Post-evis-RbM15-4-Gauteng west and Faeces-GdH35-1-Gauteng east) were indistinguishable, which suggests that either there is recirculation of the same strain through horizontal transmission across the province, or less likely, that the cattle slaughtered in these abattoirs were sourced from the same farm [9]. In addition, the two clonal complexes comprising strains from two different abattoirs and one retail outlet (Brisket-PSL1-retail/Chilled-PaQ7-1-abattoir/Perineal-PcJ28-a-abattoir and Post-evis-PdM31-1-abattoir/Pre-evis-PdK21-1-abattoir/Mince-PWNL3-2-retail) originating from the same geographic location (Gauteng north) suggest horizontal transmission and strain recirculation in Gauteng north. Recirculation of STEC could occur from carrier cattle such as super-shedders, from faecal environmental contamination including wastewater irrigations and indirectly via humans and other vectors acting as vehicles of recirculation in a geographic region [9].

The phylogenetic tree revealed that a common ancestor might exist for strains of the same sequence type in the beef production chain in South Africa. An outgroup clade comprising an isolate (Perineal swab-PdJ2-4-Gauteng north) from an abattoir hide (ST95) and three faecal isolates (Cx33-2, FaF33-1 and FaF77-2) from cattle from the feedlot (ST6353, ST11 and ST6546) was also present. The isolates belonging to these sequence types, notably ST95, had been confirmed as STEC in previous studies [20,21,22]. Interestingly, ST95 in this study also harboured four genes (UTI89_C3190, UTI89_C3191, UTI89_C3194 and UTI89_C3202) associated with uropathogenic *E. coli* (UPEC) (Supplementary Table S2) and could be related to the clonal lineage of one of the predominant clonal extraintestinal pathogenic *E. coli* (ExPEC) groups (ST131, ST69, ST95 and ST73) incriminated in human infections globally, including in the United Kingdom, Spain and France. ST95 ranked second among the most prevalent clonal ExPEC groups recovered from patients with bloodstream infections (BSI) [54]. Furthermore, the STEC O157:H7 strains predominantly belonged to ST11, which has previously been associated with diarrhoea and HUS [55].

Given the difficulty of isolating STEC from food and environmental samples, including faeces, we consider culture-based methods as a major limitation in this study. For further studies, we recommend using metagenomics to study transmission dynamics. With metagenomics, field samples can be sequenced directly, thus bypassing culture-based limitations while simultaneously increasing the opportunity for the discovery of novel pathogens [56]. Additionally, the O-antigen in most of the strains of *E. coli* could not be O-serotyped; we identify this as a limitation of the study, which requires further investigation.

In conclusion, this study provided evidence of genetic diversity in STEC strains throughout the beef production chain. The detection of *stx*_2*d*_ (*stx*_2*d*1*A*_ and *stx*_2*dB*_) and serotype O8:H19 that may cause severe disease including HC and HUS in humans is notable. The high prevalence of MDR efflux pump and *AmpC* genes constitutes a potential source of resistance genes in the southern African region, with negative impact on food security and public health. Knowledge about the prevalence of these resistance genes is crucial to curtail their dissemination in Africa. The three clonal complexes are strong evidence of horizontal transmission and recirculation of STEC strains throughout the beef production chain in Gauteng province, South Africa.

To our knowledge, this is the first study to comprehensively characterise STEC isolates recovered from the beef production chain in an area and provide evidence of horizontal transmission using WGS data. These data are valuable for hazard identification and risk assessment and for the development of intervention strategies.

## Figures and Tables

**Table 1:** Molecular and epidemiological data associated with 33 Shiga toxin-producing *Escherichia coli* isolates recovered from the beef production chain in Gauteng, South Africa.

Lab id	Sample Type *	Source	Season **	Location	Serogenotype ***	STEC	Adherence	Haemolysin
GdH35(2)	Faeces	abattoir	W	Gauteng east	H29	*stx*_2*a*_, *stx*_2*b*_	*eaeH*	*hlyE*
PaJ36(b)	perineum HS	abattoir	W	Gauteng north	H8	*stx*_1*a*_, *stx*_1*b*_, *stx*_2*a*_, *stx*_2*d*1*A*_	*eaeH*	*hlyA*, *hlyB*, *hlyC*, *hlyD*, *hlyE*
PcJ28(a)	perineum HS	abattoir	W	Gauteng north	O8:H19	*stx*_1*a*_, *stx*_1*b*_, *stx*_2*a*_, *stx*_2*b*_	*eaeH*, *ehaA*	*hlyA*, *hlyB*, *hlyC*, *hlyD*, *hlyE*
RaM39(1)	post-evisc CS	abattoir	W	Gauteng west	H19	*stx*_2*a*_, *stx*_2*b*_	*eaeH*	*hlyA*, *hlyB*, *hlyC*, *hlyD*, *hlyE*
RcM39(a)	post-evisc CS	abattoir	W	Gauteng west	H7	*stx*_1*a*_, *stx*_1*b*_, *stx*_2*a*_,	*eaeH*, *ehaA*	*hlyA*, *hlyB*, *hlyD*, *hlyE*
RaM23(1)	post-evisc CS	abattoir	S	Gauteng west	H2	*stx*_1*a*_, *stx*_1*b*_, *stx*_2*a*_,	*ehaA-*	*hlyA*, *hlyC*, *hlyD*, *hlyE*
RaN16(1)	post-evisc CS	abattoir	S	Gauteng west	H25	*stx*_1*a*_, *stx*_1*b*_	*eae*, *eaeH*, *tir*, *nleBI*, *nleB2*	*hlyA*, *hlyB*, *hlyD*, *hlyE*
PbK3(b)	pre-evisc CS	abattoir	S	Gauteng north	H19	*stx*_2*a*_, *stx*_2*b*_	*eaeH*	*hlyA*, *hlyB*, *hlyC*, *hlyD*, *hlyE*
LS 92a	faeces	farm	S	Gauteng north	H7	*stx*_2*a*_, *stx*_2*dB*_	*eaeH*	*hlyE*
CX 104(3)	faeces	farm	S	Gauteng north	H25	*stx*_2*a*_, *stx*_2*dB*_	*eae*, *eaeH*, *ehaA*, *tir*, *nleBI*	*hlyA*, *hlyB*, *hlyD*, *hlyE*
LS2 66(2)	faeces	farm	S	Gauteng north	H19	*stx*_2*a*_	*eaeH*	*hlyE*
CX 57(1)	faeces	farm	S	Gauteng north	H8	*stx*_1*a*_, *stx*_1*b*_, *stx*_2*b*_, *stx*_2*d*1*A*_	*eaeH*, *ehaA*	*hlyA*, *hlyB*, *hlyC*, *hlyD*, *hlyE*
LS2 102-3b	faeces	farm	S	Gauteng north	O9:H2/H9	*stx*_1*a*_, *stx*_1*b*_	*eae*, *tir*, *nleB2-2*	*hlyD*, *hlyE*
LS2 93c	faeces	farm	S	Gauteng north	H2	*stx*_1*a*_, *stx*_1*b*_	*eae*, *eaeH*, *tir*, *nleB2-2*	*hlyA*, *hlyB*, *hlyC*, *hlyD*, *hlyE*
CX 101(1)	faeces	farm	S	Gauteng north	H2	*stx*_1*a*_, *stx*_1*b*_	*eae*, *eaeH*, *tir*	*hlyA*, *hlyB*, *hlyD*, *hlyE*
CX 102(1)	faeces	farm	S	Gauteng north	H2	*stx*_1*a*_, *stx*_1*b*_	*eae*, *eaeH*, *tir*, *nleBI*	*hlyA*, *hlyD*, *hlyE*
CX 103(4)	faeces	farm	S	Gauteng north	H2	*stx*_1*a*_, *stx*_1*b*_	*eae*, *eaeH*, *tir*	*hlyB*, *hlyD*, *hlyE*
LS 102c	faeces	farm	S	Gauteng north	H2	*stx*_1*a*_, *stx*_1*b*_	*eae*, *eaeH*, *tir*	*hlyB*, *hlyD*, *hlyE*
CX 8(4)	faeces	farm	S	Gauteng north	0	*stx*_1*a*_	*eaeH*, *eaeX*	*0*
FAF 33(1)	faeces	farm-abattoir	S	Gauteng north	H7	*stx*_2*b*_	*eae*, *eaeH*, *tir*, *nleB2*	*hlyD*, *hlyE*
FAF 8(8)	faeces	farm-abattoir	S	Gauteng north	H25	*stx*_2*a*_, *stx*_2*dB*_	*eae*, *eaeH*, *ehaA*, *tir*	*0*
FAF 85(1)	faeces	farm-abattoir	S	Gauteng north	H8	*stx*_1*a*_, *stx*_1*b*_, *stx*_2*b*_, *stx*_2*d*1*A*_	*eaeH*	*hlyA*, *hlyB*, *hlyC*, *hlyD*, *hlyE*
FAF 68(1)	faeces	farm-abattoir	S	Gauteng north	H2	*stx*_1*a*_, *stx*_1*b*_, *stx*_2*a*_, *stx*_2*dB*_	*eaeH*, *saa*	*hlyA*, *hlyB*, *hlyC*, *hlyD*, *hlyE*
FAF 68(a)	faeces	farm-abattoir	S	Gauteng north	H2	*stx*_1*a*_, *stx*_1*b*_, *stx*_2*a*_,	*eaeH*	*hlyA*, *hlyB*, *hlyC*, *hlyD*, *hlyE*
FAF 51(1)	faeces	farm-abattoir	S	Gauteng north	H25	*stx*_1*a*_, *stx*_1*b*_	*eae*, *eaeH*, *tir*, *nleB2*	*hlyA*, *hlyB*, *hlyD*, *hlyE*
FA3 9B(1)	faeces	farm-env	S	Gauteng north	H29	*stx*_2*b*_, *stx*_2*dB*_	*eaeH*	*hlyE*
FA3 9B(3)	faeces	farm-env	S	Gauteng north	H29	*stx*_2*b*_, *stx*_2*d*1*A*_, *stx*_2*dB*_	*eaeH*	*hlyE*
SSL5(1)	biltong	retail	S	Gauteng north	H29	*stx_2d1_*	*eaeH*	*hlyE*
WWL2(2)	boerewors	retail	S	Gauteng north	O8:H19	*stx*_2*a*_, *stx*_2*dB*_	*eaeH*	*hlyA*, *hlyB*, *hlyC*, *hlyD*, *hlyE*
WWL2(3)	boerewors	retail	S	Gauteng north	O8:H19	*stx*_2*a*_, *stx*_2*dB*_	*eaeH*, *saa*	*hlyA*, *hlyC*, *hlyD*, *hlyE*
KEL2(3)	boerewors	retail	A	Gauteng north	H19	*stx*_2*a*_	*eaeH*	*hlyE*
PSL1(3)	brisket	retail	A	Gauteng north	H12	*stx*_2*a*_, *stx*_2*dB*_	-	*hlyE*
PSL1(4)	brisket	retail	A	Gauteng north	O8:H19	*stx*_1*a*_, *stx*_1*b*_, *stx*_2*a*_, *stx*_2*dB*_	*eaeH*, *saa*	*hlyA*, *hlyB*, *hlyC*, *hlyD*, *hlyE*

* Sample type: CS—carcass swab, HS—hide swab; ** Season: S—summer, W—winter, A—autumn; *** Serogenotype: For isolates that were classified as O-nontypeable (ONT), only the flagellar type was listed.

**Table 2:** MLST clonal complexes found in 85 Shiga toxin-producing *Escherichia coli* isolates recovered along the beef chain in Gauteng, South Africa.

Clonal Complex	Sample	Source	Location *	ST ^†^	Serogenotype	*stx*-Subtypes
5 matching loci	Post-evisceration-RbM15-4	abattoir	GW	ST1049	H10	-
	Pre-evisceration-PdK13-2	abattoir	GN	ST155	H21	-
	Faeces-GdH35-1	abattoir	GE	ST58	O8:H30	-
5 matching loci	Brisket-PSL1-4	retail	GN	ST201	O8:H19	*stx*_1*a*_, *stx*_1*b*_, *stx*_2*a*_, *stx*_2*dB*_
	Chilled-PaQ7-1	abattoir	GN	ST162	O8:H19	-
	Perineal-PcJ28-a	abattoir	GN	ST469	O8:H19	*stx*_1*a*_, *stx*_1*b*_, *stx*_2*a*_, *stx*_2*b*_
6 matching loci	Post-evisceration-PdM31-1	abattoir	GN	ST1145	H16	-
	Pre-evisceration-PdK21-1	abattoir	GN	ST10	O6:H12	-
	Mince-PWNL3-2	retail	GN	ST1141	H4	-

* Location: Gauteng west—GW, Gauteng north—GN, Gauteng east—GE. ^†^ Sequence type.

**Table 3:** Occurrence of 66 genes that code for antimicrobial resistance in 85 STEC isolates from the beef production chain in Gauteng, South Africa.

Antimicrobial Class	Resistance Genes	No. of Isolates Positive for AMR Genes	Antimicrobial Compounds
Aminoglycosides	*aph* (3″)-Ib	8	Aminoglycoside
	*aph* (6)-Id	8	Aminoglycoside
	*aadA*	4	Aminoglycoside
	*aadA2*	2	Aminoglycoside
	*aadA3*	3	Aminoglycoside
	*aadA4*	1	Aminoglycoside
	*kdpE*	83	Aminoglycoside
Amphenicols	*cmlA6*	1	Amphenicols
	*floR*/chloramphenicol	3	Amphenicols
Beta-lactam/beta-lactamase-inhibitor	*ampC*	83	Cephalosporin, penam
	*TEM-1*	2	Penam, monobactam, penem, cephalosporin
	*TEM-150*	1	Penam, monobactam, penem, cephalosporin
Fluoroquinolones	*patA*	84	Fluoroquinolone
Glycopeptides	*bacA*	84	Peptide
	*eptA*/*PmrC*	85	Peptide
	*pmrF*	85	Peptide
	*ugd*/*pmrE*	73	Peptide
	*yojI*	84	Peptide
Macrolide	*mphB*	4	Macrolide
Multidrug (MDR) efflux pumps	*CRP*	85	Penam, macrolide, fluoroquinolone
	*acrA*	85	Tetracycline, glycylcycline, rifamycin, phenicol, penam, cephalosporin, fluoroquinolone, disinfecting agents and antiseptics
	*emrE*	63	Macrolide
	*mdfA*	84	Disinfecting agents and antiseptics, tetracycline
	*H-NS*	83	Macrolide, tetracycline, penam, cephalosporin, fluoroquinolone, cephamycin
	*acrB*	82	Tetracycline, glycylcycline, rifamycin, phenicol, penam, cephalosporin, fluoroquinolone, disinfecting agents and antiseptics
	*acrD*	82	Aminoglycoside
	*acrE*	82	Penam, cephamycin, fluoroquinolone, cephalosporin
	*acrF*	79	Penam, cephamycin, fluoroquinolone, cephalosporin
	*acrS*	80	Tetracycline, cephamycin, glycylcycline, rifamycin, phenicol, penam, cephalosporin, fluoroquinolone, disinfecting agents and antiseptics
	*baeR*	85	Aminocoumarin, aminoglycoside
	*baeS*	85	Aminocoumarin, aminoglycoside
	*cpxA*	84	Aminocoumarin, aminoglycoside
	*emrA*	85	Fluoroquinolone
**Antimicrobial Class**	**Resistance Genes**	**Number of Resistant Isolates**	**Antimicrobial Compounds**
Multidrug (MDR) efflux pumps	*emrB*	85	Fluoroquinolone
	*emrK*	83	Tetracycline
	*emrR*	85	Fluoroquinolone
	*emrY*	79	Tetracycline
	*evgA*	84	Penam, tetracycline, macrolide, fluoroquinolone
	*evgS*	78	Penam, tetracycline, macrolide, fluoroquinolone
	*gadW*	83	Penam, macrolide, fluoroquinolone
	*gadX*	81	Penam, macrolide, fluoroquinolone
	*marA*	85	Tetracycline, glycylcycline, rifamycin, phenicol, penam, cephalosporin, cephamycin, penem, monobactam, carbapem, fluoroquinolone, disinfecting agents and antiseptics
	*mdtA*	84	Aminocoumarin
	*mdtB*	84	Aminocoumarin
	*mdtC*	84	Aminocoumarin
	*mdtE*	82	Fluoroquinolone, macrolide, penam
	*mdtF*	81	Fluoroquinolone, macrolide, penam
	*mdtH*	84	Fluoroquinolone
	*mdtK*	66	Fluoroquinolone
	*mdtM*	83	Nucleoside, lincosamide, fluoroquinolone, phenicol, disinfectant agents and antiseptics
	*mdtN*	85	Nucleoside, disinfectant and antiseptics
	*mdtO*	82	Nucleoside, disinfectant and antiseptics
	*mdtP*	83	Nucleoside, disinfectant and antiseptics
	*msbA*	85	Nitroimidazole
	*tolC*	85	Peptide, aminoglycoside, tetracycline, aminocoumarin, penem, phenicol, fluoroquinolone, carbapem, macrolide, disinfecting agents and antiseptics, cephalosporin, glycylcycline, rifamycin, cephamycin
	*vgaC*	6	Streptogramin, pleuromutilin, streptogramin A, lincosamide
Phosphonics	*FosA7*	4	Phosphonic acid
	*mdtG*	84	Phosphonic acid
Sulfonamides	*sul1*	5	Sulphonamide
	*sul2*	7	Sulphonamide
	*sul3*	1	Sulphonamide
Tetracyclines	*tet*(*A*)	2	Tetracycline
	*tet*(*C*)	6	Tetracycline
	*tet*(*D*)	1	Tetracycline
Trimethoprim-derivatives	*dfrA12*	4	Diaminopyrimidine
	*dfrA15*	3	Diaminopyrimidine

## Data Availability

The data presented in this study are openly available in [National Center for Biotechnology Information] at https://account.ncbi.nlm.nih.gov/?back_url=https%3A%2F%2Fdataview.ncbi.nlm.nih.gov%2F, reference number [PRJNA706921], accessed on 20 July 2024.

## Supplementary Materials

The following supporting information can be downloaded at: https://www.mdpi.com/article/10.3390/pathogens13090732/s1 Table S1: Information on 85 isolates used in this study from the beef production chain in Gauteng, South Africa; Table S2: Occurrence of 552 VFDB annotated genes in the 85 STEC genome assemblies.; Table S3: Occurrence of 66 genes that code for antimicrobial resistance (AMR) in 85 STEC isolates from the beef production chain in Gauteng, South Africa
